# Adhesion and Growth of Neuralized Mouse Embryonic Stem Cells on Parylene-C/SiO_2_ Substrates

**DOI:** 10.3390/ma14123174

**Published:** 2021-06-09

**Authors:** Alan F. Murray, Evangelos Delivopoulos

**Affiliations:** 1 School of Engineering, University of Edinburgh, Edinburgh EH9 3FB, UK; Alan.Murray@ed.ac.uk; 2 School of Biological Sciences, University of Reading, Reading RG6 6DH, UK

**Keywords:** parylene-C, mESCs, neuron

## Abstract

Neuronal patterning on microfabricated architectures has developed rapidly over the past few years, together with the emergence of soft biocompatible materials and tissue engineering scaffolds. Previously, we introduced a patterning technique based on serum and the biopolymer parylene-C, achieving highly compliant growth of primary neurons and astrocytes on different geometries. Here, we expanded this technique and illustrated that neuralized cells derived from mouse embryonic stem cells (mESCs) followed stripes of variable widths with conformity equal to or higher than that of primary neurons and astrocytes. Our results indicate the presence of undifferentiated mESCs, which also conformed to the underlying patterns to a high degree. This is an exciting and unexpected outcome, as molecular mechanisms governing cell and ECM protein interactions are different in stem cells and primary cells. Our study enables further investigations into the development and electrophysiology of differentiating patterned neural stem cells.

## 1. Introduction

Tissue architecture is one of the most important parameters dictating organ function, where it ranges from simple (e.g., kidney) to very complex (e.g., central nervous system). Cell patterning as a research area encompasses multiple techniques to direct cell placement and growth on a variety of 2D and 3D substrates [1]. The use of microcontact printing [2], stencils [3], laser ablation [4] and self-assembled monolayers [5] are invaluable techniques in the fields of tissue engineering, as they facilitate organoid development in biocompatible scaffolds [6,7]. Furthermore, cell patterning has enabled biomaterial evaluation [8] and unlocked new modalities in probing specific tissue development and function [9].

Our lab developed a cell patterning technique based on the polymer parylene-C, which was deposited and patterned on a silicon oxide (SiO_2_) background. Parylene-C is hydrophobic due to its low surface energy and is often used as an encapsulant for implantable electrodes and devices [10,11]. In contrast, SiO_2_ is hydrophilic and is the most commonly used substrate in microfabrication. It has been used extensively in neuronal patterning [12] and as a dielectric and encapsulant for in vivo neuronal probes [13]. Proteins that adsorb onto parylene-C and SiO_2_ surfaces have radically different conformational profiles [14]. This is due to the drastically different surface energies of parylene-C and SiO_2_. Therefore, cell adhesion and patterning on a parylene-C/SiO_2_ substrate can be achieved via immersion of the substrate in serum, as conformationally distinct fibronectin (Fn) and albumin protein layers assemble on the parylene-C and SiO_2_ surfaces [14]. Specifically, Delivopoulos et al. produced patterned cultures of rat hippocampal neurons and astrocytes on bands of parylene-C that were patterned on SiO_2_ substrates via photolithography [15] or laser ablation [16]. In subsequent studies, the method was optimised by: (a) reducing the parylene-C thickness from 100 to 10 nm [17] to enable the use of this technique in capacitive coupling recordings, (b) controlling the pattern permittivity to neurons and astrocytes via UV irradiation [18] and (c) preserving cellular conformity to patterns via antimitotic drug treatment [19]. The parylene-C patterning technique was also applied to other cell types, such as the human teratocarcinoma cell line [20], achieving pattern fidelity with single-cell resolution [21]. Recent studies even demonstrated Ca^2+^ transients in astrocytic networks patterned in parylene-C trenches [22,23].

Parylene-C has been used as a stencil to pattern murine embryonic stem cells (ESCs) in co-cultures. Wright et al. patterned mouse ESCs that were co-cultured with NIH-3T3 fibroblasts and AML-12 hepatocytes [24]. Similarly, Jinno et al. generated dynamic co-cultures of mESCs and human umbilical vein endothelial cells (HUVECs), murine epithelial ameloblast-lineage cells (ALCs) and NIH-3T3 fibroblasts by sequentially peeling parylene-C stencils [25]. Even though such studies are valuable in broader investigations of cellular interactions in controlled microenvironments, the requirement for mechanical manipulation of a thin polymer layer limits the complexity of possible pattern geometries and the minimum spatial resolution of the patterns, while disrupting long-term recordings. Microfabrication techniques offer substantial flexibility in manipulating the topography of patterns, which have been used in a variety of investigations, including the adipogenic [26] and myocardial [27] differentiation of mesenchymal stem cells (MSCs), as well as the stemness [28] and function [29] regulation of MSCs. Soft lithography has also allowed for the generation of 3D platforms that have been used in the study of stem cell differentiation, cancer cell migration and endothelium modeling [30]. There, scaffolds had a substantial impact on tissue engineering, as they mimic the stem cell microenvironment [31] and enable both fundamental studies but also the clinical translation of established technologies. However, despite the significant expansion of the cell patterning field, few studies have demonstrated high-fidelity mESC-derived neuronal patterning via microfabrication-compatible techniques.

In this work, our motivation was to extend the parylene-C patterning method to neuralized cells derived from mouse embryonic stem cells. We prepared micropatterns of parylene-C of different geometries on SiO_2_ and cultured neuralized mESCs, as well as primary hippocampal neurons and astrocytes. Our results reveal high conformity of neuralized stem cells to the underlying parylene-C patterns, with the geometry being an important factor, where the narrower stripes had significantly higher indexes of conformity. Furthermore, serum activation of the substrates increased the adherence of these cells to the parylene-C patterns. Primary neurons and astrocytes cultured on patterns of similar geometry, also adhered predominantly on the paryelne-C and had similar conformity indexes to the stem cell group of cells.

## 2. Materials and Methods

### 2.1. Ethics Statement

All protocols and procedures involving animals were carried out with the approval of the Home Office UK and in strict adherence to the Animals (Scientific Procedures) Act 1986 under license PPL 60/3395 “Physiological studies of neurodegenerative disease”.

### 2.2. Maintenance and Culture of Mouse Embryonic Stem Cells

The mouse embryonic stem cell (mESC) line CGR8 (derived from Mus musculus, strain 129) were incubated at 37 °C, 5% CO_2_, in gelatine coated flasks, in a media composed of DMEM, supplemented with 10% fetal calf serum (FCS) (Gibco Industries, Inc., Langley, OK, USA), 1% penicillin/streptomycin, 1% l-glutamine (Life Technologies, Paisley, UK), 100 μM 2-mercaptoethanol and LIF (Leukemia Inhibitory Factor) (1000 units/mL) (Sigma Aldrich, Gillingham, UK). Cells were passaged every 2 days and assessed daily for confluence.

### 2.3. Embryonic Stem Cell Neuralisation

Mouse ESCs were differentiated into neurons by adapting a mass suspension protocol from Peljto et al. [32]. On day 0, mES cells were seeded on non-tissue-culture-treated Petri dishes (50,000 cells/mL) and allowed to aggregate into embryoid bodies (EB) in ADFNK media (ADMEM/F12:Neurobasal medium (1:1), 10% Knockout Serum Replacement, 1% penicillin/streptomycin, 1% l-glutamine, 100 μM 2-mercaptoethanol), without LIF. Fresh media were added on day 2 and day 5 of differentiation. On day 2, 1 μM RA and 1 μM purmorphamine were supplemented into the media. On day 6, EBs were collected, washed with PBS and resuspended in trypsin/EDTA for 10 min at 37 °C. This process breaks down EBs and releases individual cells into the suspension. Trypsin was inactivated with aggregation medium and cells were centrifuged at 180× *g* for 5 min. The supernatant was then aspirated. Cells were resuspended in an aggregation medium and then filtered through a 70 μm cell strainer on top of a 50 mL centrifuge tube in order to remove any large aggregates and the matrix. The cell density in the suspension was counted in a hemocytometer. Individual cells were seeded at 150 cells/mm^2^ on serum activated and control patterns and incubated for 5 days in vitro (DIV) at 37 °C, 5% CO_2_ in ADFNB media (ADMEM/F12:Neurobasal (1:1), 1× B-27 supplement, 1% penicillin/streptomycin, 1% l-glutamine, 100 μM 2-mercaptoethanol). Media were exchanged 2 days after plating.

### 2.4. Primary Cell Culture

We adapted the gradient cell isolation protocol presented by Brewer et al. [33]. We harvested and mechanically dissociated hippocampal cells from P1–P7 (postnatal) Sprague Dawley rats. Cells were plated at 150 cells/mm^2^ in Neurobasal/B27 medium containing l-glutamine (0.5 mM), 1% penicillin/streptomycin, bFGF (10 ng/mL) and brain-derived neurotrophic factor (BDNF) (20 ng/mL) to promote neuronal viability and health. Cultures were incubated at 37 °C and 5% CO_2_ for 1, 2 and 3 weeks. Old media were exchanged with fresh media at 3 days after plating. After the first week, the media were exchanged every 2 days, with 5 µM cytosine arabinoside (AraC) being added in every other exchange to control glia division.

### 2.5. Immunocytochemistry and Cell Labeling

Samples were fixed for 25 min in 3.7% PFA, blocked and permeabilised in 20% normal goat serum (NGS) in 0.05% Triton-X-100 in DPBS for 1.5 h at room temperature. Primary antibodies were applied for 1.5 h at room temperature or overnight at 4 °C and secondary antibodies were applied for 2 h at room temperature. Nuclei were counterstained with Hoechst 33342 (1:25,000) for 10 min at room temperature. All washes were in DPBS. Antibodies used were: anti-b-III-tubulin chicken IgY (Abcam, Cambridge, UK), anti-GFAP rabbit IgY (Abcam), anti-chicken Alexa Fluor 594, anti-rabbit Alexa Fluor 488 and Cell Tracker Green (CMFDA C7025). Samples were mounted using a Vectashield mounting medium and imaged using a Leica Confocal Microscope (Zeiss, Birmingham, UK) to take images covering areas of 4 mm^2^. We imaged at the excitation wavelengths of the antibodies (590 nm and 495 nm) and the reflection of each pattern.

### 2.6. Pattern Fabrication, Sterilisation and Activation

**Figure 1:**
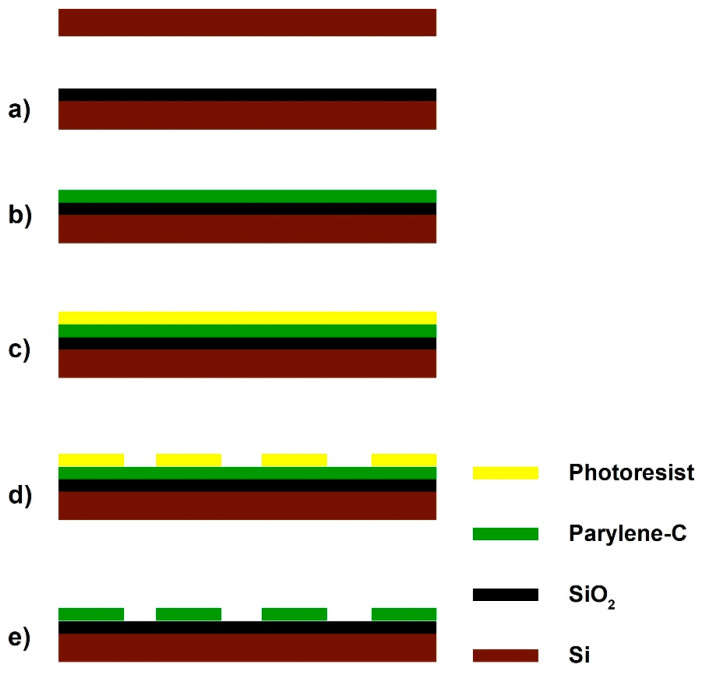
Fabrication flow of a parylene-C pattern: (**a**) growth of a 200 nm SiO_2_ layer via thermal oxidation; (**b**) deposition of a 10–20 nm parylene-C layer; (**c**) spin coating of a positive photoresist, followed by a 60 s soft bake at 90 °C; (**d**) development of exposed photo-resist after a 60 s post-exposure bake at 110 °C; (**e**) etching of unprotected parylene-C in Plasma-Therm and rinsing the residual photoresist.

A 200 nm layer (inspected using Nanospec) of SiO_2_ was grown on silicon (H2 1.88 sccm and O_2_ 1.25 sccm, 950 °C for 40 min), as shown in Figure 1. Parylene-C, 100 nm thick, was deposited at room temperature on the oxidised wafers via a Labcoter 2 Parylene Deposition Unit (Model PDS2010) at a rate of 1.298 nm/mg of dimer. Wafers were then coated with hexamethyldisilazane (HMDS) and the positive photoresist Rohm and Hass SPR350-1.2 (1 mm theoretical thickness), followed by a 60 s soft bake at 90 °C. An Optimetrix 8605 5x reduction stepper was used, together with a previously created mask to print stripe patterns on each wafer. After a 60 s post-exposure bake at 110 °C, wafers were developed in Microchem MF-26A. Unprotected parylene-C was etched off in the Plasma-Therm (90 s, 50 mTorr chamber pressure, 50 sccm O_2_, 500W RF power, etch rate was approximately 100 nm/min) to reveal the SiO_2_ underneath. Complete etching was verified with Nanospec. Residual photoresist was removed with acetone. Wafers were cut with a DISCO DAD 800 Dicing Saw (spindle speed 30000 rpm, feed speed 7 mm/s), rinsed with distilled ionised (DI) water and blown dry with N_2_. Figure 1 summarises this process flow. Five stripe patterns were fabricated with dimensions: 2 mm length and 5 μm, 10 μm, 20 μm, 30 μm and 40 μm width. The relative percentage areas of each parylene-C pattern compared to the total surface was 2.5%, 5%, 10%, 15% and 20%, respectively. Data from different pattern geometries were analysed together. Equal *N* numbers of different geometries were tested. Individual substrates were cleaned in piranha acid (30% H_2_O_2_, 98% H_2_SO_4_ in a 5:3 ratio), sterilised in pencilin/streptomycin for 1 h and activated via a 3 h immersion in horse serum (GIBCO, Loughborough, UK). Control samples were not immersed in serum. Ethanol-cleaned glass coverslips were coated with 0.5 mL of poly-d-lysine solution (50 mg/mL) and cultured with a sample of the primary neuronal population within each harvest. This was done to ensure that the primary neurons and astrocytes were abundant and morphologically healthy (see Supplementary Figure S1).

### 2.7. Statistical Analysis

The conformity of the cells to the parylene-C stripes was assessed using the conformity index (CI). Using the Leica Lite software, we selected each parylene-C stripe and calculated the histograms of the pixel intensity for both green and red channels. We chose a threshold for the image corresponding to the histograms that included the maximum amount of biological data possible (axons, dendrites and cell somata), while eschewing artifacts and minimising noise. The histogram area above the threshold value was considered as the quantity of astrocytic/cellular (green) or neuronal (red) pixels present on the parylene-C.

We repeated this process for the entire image to acquire the total quantity of astrocytic, cellular and neuronal pixels on the substrate. These two quantities were divided to derive the percentage of pixels on the parylene-C stripes. Finally, percentages were normalised with regard to different geometries by dividing with the percentage area of each pattern (2.5%, 5%, 10%, 15% and 20%). Therefore, a CI of 1 would signify a random culture. As the culture becomes perfectly patterned, CI values approach ∞ (infinity). For example, the conformity index for astrocytes on a 10% stripe pattern would be

(1) $$Conformity\;Index=\frac{Total\;astrocyte\;pixels\;on\;parylene-C}{Total\;astrocyte\;pixels\;on\;the\;substrate*10\%}$$

Conformity indexes from multiple images were statistically analysed using Minitab software (version 19.1.1.) with a 2-way ANOVA (general linear model) to ascertain whether there was a significant difference between the means of CIs of different factors. Bonferroni pairwise comparisons were also conducted.

Model used: *y* = *b*_0_ + *bx* + *e*, *y*—set of outcome variables (conformity indexes), *x*—set of covariates, *b*_0_—set of intercepts, *b*—set of coefficients for each covariate and *e*—error.

The two factors examined for stem cell cultures were: (a) geometry (5 μm, 10 μm, 20 μm, 40 μm) and (b) treatment (serum, no serum). The data for neurons (b-III-tubulin, red channel) and all live cells (Cell Tracker Green, green channel) were analysed separately. Statistical significance was assumed when *p* < 0.05. For primary cell cultures the three factors were: (a) geometry (10 μm, 20 μm, 30 μm, 40 μm), (b) treatment (serum, no serum) and (c) DIV (7, 14, 21). The data for neurons (b-III-tubulin, red channel) and astrocytes (GFAP, green channel) were analysed separately.

## 3. Results

### 3.1. Stem Cell Guidance on Parylene-C Patterns

**Figure 2:**
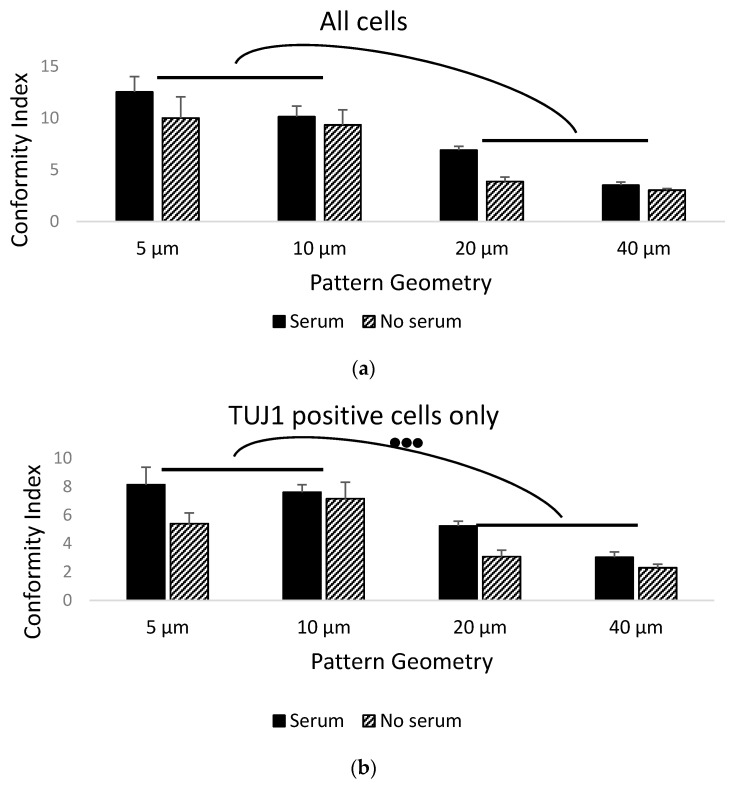
Bar graphs of the conformity index of: (**a**) all cells patterned on parylene-C and (**b**) TUJ1^+^ cells patterned on paryelene-C. Black bars denote the average CIs of serum-treated samples (*N* = 8), whereas stripped bars denote the average CIs of untreated samples (*N* = 4). Results highlight the importance of serum in mESC and TUJ1^+^ cell patterning, as well as narrower geometries being superior at guiding cellular migration and growth. Three dots denote significance at *p* < 0.001 between the depicted geometry groupings. Error bars represent the standard error of the mean (SEM).

We neuralized and patterned the mESC line CGR8 on serum-activated parylene-C stripes on a SiO_2_ background. The bar graphs of the conformity indexes for all cells (Figure 2a) and for the TUJ1^+^ cells (Figure 2b) illustrate the differences in cell/neuronal conformity to the underlying patterns between the controls and serum-treated samples, as well as the patterns of different geometries. These differences were confirmed using two-way ANOVA as being statistically significant (*p* < 0.05 for serum vs. control and *p* < 0.001 for different geometries). The serum treatment greatly enhanced the cell conformity, which was an effect seen in our initial publication on parylene-C patterning [15]. The average CIs for serum-treated samples were between 3.51 ± 0.31 (40 μm patterns) and 12.52 ± 1.48 (5 μm patterns), whereas, for the control samples, the average CIs were between 3.02 ± 0.15 (40 μm patterns) and 9.99 ± 2.07 (5 μm patterns). When considering the TUJ1^+^ cells only, the average CIs for the serum-treated samples were between 3.04 ± 0.37 (40 μm patterns) and 8.14 ± 1.23 (5 μm patterns), whereas, for the control samples, the average CIs were between 2.3 ± 0.25 (40 μm patterns) and 5.39 ± 0.75 (5 μm patterns). Similarly, geometry seemed to be an important factor in patterning undifferentiated and neuralized stem cells. This contrasts with our prior results with primary cells [15], where pattern geometry did not significantly affect neuronal and astrocyte conformity to patterns when substantially wider parylene-C stripes (20–100 μm) were used. In this study, stem cells seemed to follow the parylene-C better for narrower stripes.

**Figure 3:**
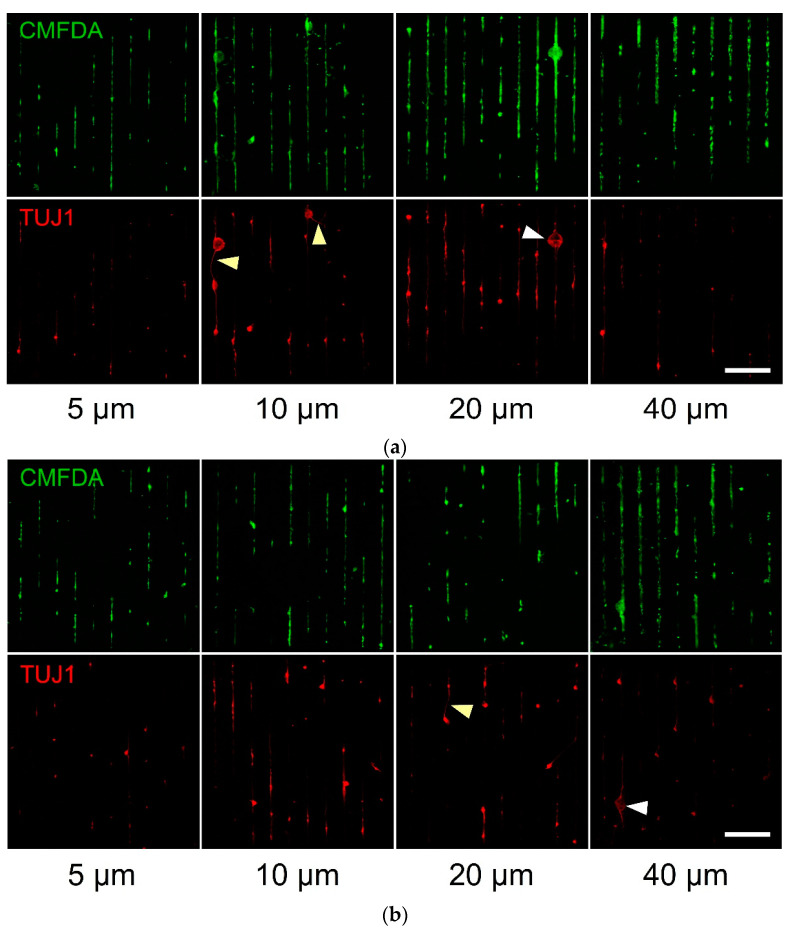
Patterned stem cell culture examples on parylene-C stripes of different geometries (width of parylene-C stripes below each column). The CMFDA dye (green channel) stained all the cells, whereas the TUJ1 antibody stained neuronal cells only (red channel): (**a**) patterns treated with serum and (**b**) untreated patterns. White arrowheads point to neuronal clusters and yellow arrowheads to extended neurites. Scale bar is 500 μm.

The following culture photomicrographs illustrate the cellular and neuronal conformity to the parylene-C between the serum-treated (Figure 3a) and control (Figure 3b) patterns of different geometries. We noticed higher cellular content in the serum-treated patterns (Figure 3a) than in the control ones (Figure 3b). This again confirmed our prior results, where primary neurons and astrocytes extended processes on the serum-treated patterns. In this study, neuralized stem cells formed clusters (Figure 3a, white arrowheads); however, there was a minimal extension of neurites (Figure 3a, yellow arrowheads), as these neurons are not yet fully mature. Control patterns had minimal neuronal content with few clusters and even fewer processes (Figure 3b, white and yellow arrowheads).

There was also a population of cells that were not TUJ1^+^ (Figure 3, cells that were CMFDA^+^ and TUJ1^−^). These could be undifferentiated stem cells or cells of ectoderm lineage that did not yet express βIII-tubulin.

### 3.2. Primary Cell Patterning with Parylene-C

**Figure 4:**
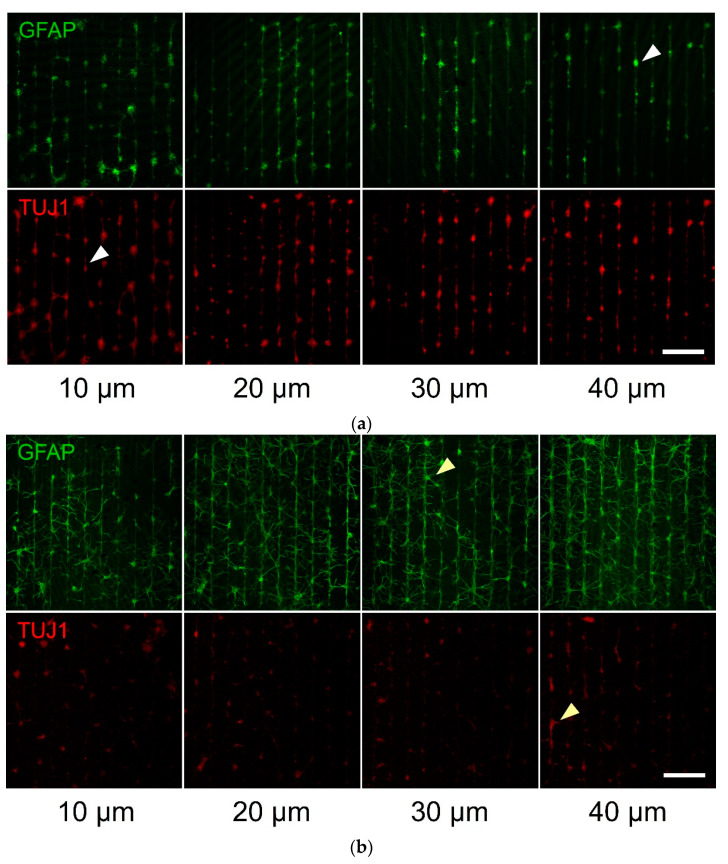

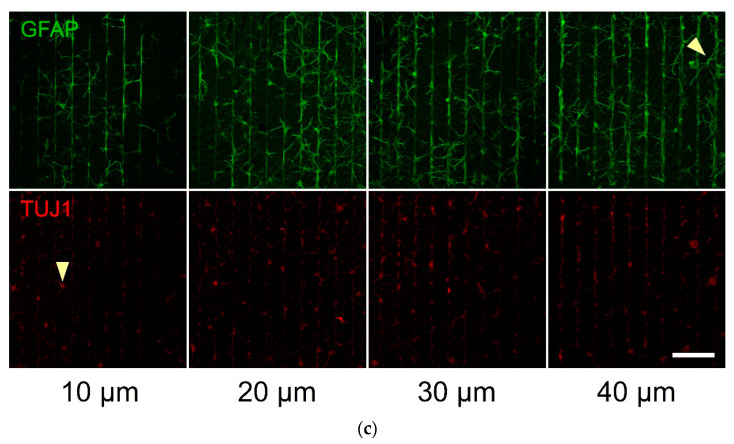
Patterned primary culture examples of hippocampal neurons and astrocytes on parylene-C stripes of different geometries (width of parylene-C stripes below each column). The GFAP antibody (green channel) stained the astrocytes, whereas the TUJ1 antibody only stained the neuronal cells (red channel): (**a**) 7 DIV culture, (**b**) 14 DIV culture and (**c**) 21 DIV culture. White arrowheads point to cells localised on the parylene-C stripes, while yellow arrowheads point to processes bridging neighbouring stripes. Scale bar is 500 μm.

**Figure 5:**
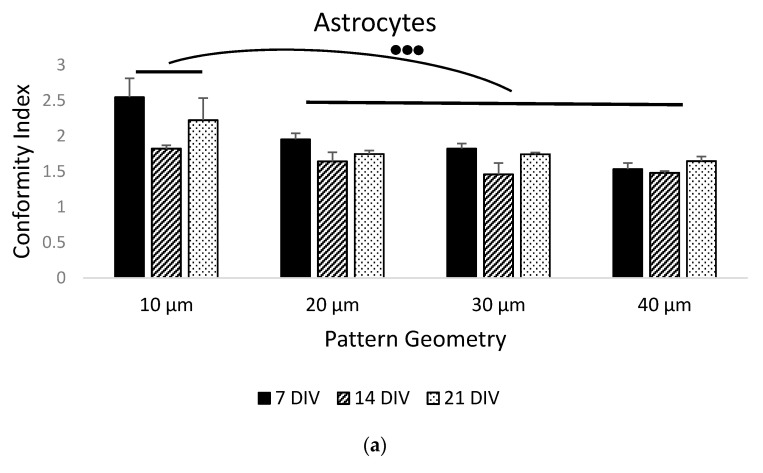

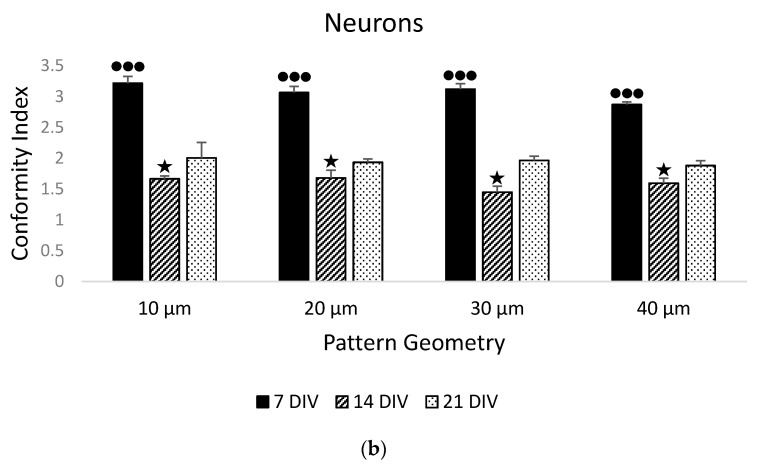
Bar graphs of the conformity index of: (**a**) hippocampal astrocytes (GFAP^+^) and (**b**) hippocampal neurons (TUJ1^+^) patterned on parylene-C. Black bars denote the average CIs of the 7 DIV culture samples (*N* = 3), striped bars denote the average CIs of the 14 DIV culture samples (*N* = 3) and dotted bars denote the average CIs of the 21 DIV culture samples (*N* = 4). Results highlight the loss of conformity to the patterns as the culture time progressed, as well as the narrowest geometry (10 μm) being superior in guiding astrocytic migration and growth. Three dots denote significance at *p* < 0.001 between respective groups. The star denotes significance at *p* < 0.001 between 14 and 21 DIV. Error bars represent the standard error of the mean (SEM).

In order to test the fabricated substrates, we also cultured primary hippocampal neurons and astrocytes on serum-treated parylene-C patterns. We monitored the adherence and growth of neurons and astrocytes over the course of three weeks. Cells were strictly localised on the stripe patterns during the first week (DIV 7, Figure 4a, white arrowheads), and gradually extended the processes to neighboring stripes over the course of two weeks (DIV 14, Figure 4b and DIV 21, Figure 4c, yellow arrowheads). This was reflected by the declining CIs in Figure 5 and was confirmed as statistically significant using a two-way ANOVA for both astrocytes (CI means—DIV7: 1.97, DIV14: 1.60, DIV21: 1.84, *p* < 0.05) and neurons (CI means—DIV7: 3.06, DIV14: 1.6, DIV21: 1.94, *p* < 0.001). As with prior results, geometry was not a significant factor in neuronal patterning (CI means—10 μm patterns: 2.29, 20 μm patterns: 2.22, 30 μm patterns: 2.17, 40 μm patterns: 2.13, *p* = 0.445, *N* = 9). With regard to astrocytes, there was a statistically significant difference between the 10 μm pattern (CI mean: 2.18) and the 20 μm (CI mean: 1.77), 30 μm (CI mean: 1.68) and 40 μm (CI mean: 1.58) patterns (*p* < 0.01).

## 4. Discussion

In this study, we applied the parylene-C cell patterning technique to growing and differentiating mouse embryonic stem cells (CGR8 cell line) on stripes of variable widths. Through a series of cultures, we illustrated that both neuralized cells (TUJ1^+^) and undifferentiated stem cells (CMFDA^+^/TUJ1^−^) aligned faithfully to the underlying patterns. As shown in prior studies, after serum immersion, albumin, fibronectin, vitronectin and other cell adhesive proteins adsorb onto parylene-C and SiO_2_ surfaces [15]. Anchored proteins on the two substrates have different conformations, generating the preferential adhesion of primary cells to parylene-C [14]. It is surprising that stem cells respond to adsorbed cell adhesion proteins on paryelne-C considering that their integrin receptors are strongly dependent on their niche and oriented towards facilitating stem cell interactions. For example, the main integrin receptor of neural stem cells from the mouse subventricular zone is α6β1 and responds to laminin [34], whereas the attachment of primary neonatal rat astrocytes to vitronectin is primarily mediated by integrins αvβ5 and α8β1 [35]. Furthermore, in cell maintenance protocols, mESC attachment is mediated via gelatin, not vitronectin. It is possible that that integrin receptors on the remaining undifferentiated mESCs respond to the adsorbed protein layer on the parylene-C. Alternatively, differentiating neurons may be mature enough to attach to parylene-C via the usual receptor–protein interactions and attract stem cells onto the stripes via paracrine signaling and cell–cell interactions. If we consider the increased cellular content and the extension of neuronal processes in serum-treated samples (Figure 3a), it is certain that differentiating neurons adhered optimally to the parylene-C. This result expands our patterning technique and increases its flexibility.

Geometry was a significant factor in patterning both undifferentiated and neuralized mESCs with parylene-C, as there was a statistically significant difference in the compliance index between the narrower (5 μm and 10 μm) and wider stripes (20 μm and 40 μm) (Figure 2a,b). In prior studies, when we examined the effect of stripe width (5–20 μm) on primary neuron and astrocyte patterning, we observed mixed results. Neurons concentrated and complied with narrower stripes better, regardless of the stripe length. On the other hand, astrocytes preferred wider stripes [17]. Similarly, Corey et al. [36] and Lauer et al. [37] found strong correlations between neuronal compliance to grid patterns and node diameter, internodal distance, and stripe width. Our hypothesis is that geometries containing features between 5 and 40 μm influence neuronal, astrocyte and stem cell growth, as these topographical cues are comparable to the size of the cells, enabling tactile interactions, which act synergistically to biochemical signals.

Primary hippocampal neurons and astrocytes were also patterned on parylene-C stripes of comparable widths (10–40 μm) over the course of 3 weeks. As expected, there was a sharp decline in neuronal conformity between the first and two final weeks. Despite the use of AraC, there was astrocyte division and expansion into neighbouring stripes, which promoted off-pattern neuronal growth. Nonetheless, all CIs remained above 1.0, which signifies a minimum degree of neuronal and astrocyte patterning, even in the DIV 14 and 21 cultures. We observed that this synergy between neuron and astrocyte patterning was crucial, particularly in longer cultures. Therefore, mESC-derived neuronal patterning for extended periods may be challenging in the absence of astrocytes.

During development, neuronal migration and axonal and dendritic guidance are crucial processes in the establishment of the connectome. Many nervous system disorders are associated with failures in neural patterning and changes in the structural and functional connectivity in the brain. In vitro cell-patterning techniques can shed light on neurodevelopmental processes and highlight strategies for combating these disorders, either early in development or after nervous system maturation. The parylene-C patterning technique is simple and cost effective with high efficiency. In this work, we introduced the possibility of patterning differentiating stem cells that were directed towards a specific neuronal lineage. With our patterning technique, we can engineer complex neural architectures composed of different neuronal subtypes, neurotransmitter systems, and hence, activity profiles. These engineered networks are already being used in pharmacological research, reducing and replacing animal testing. The introduction of stem cells into refined cell patterning tehcniques paves the way for neural tissue replacement after stroke and spinal cord injury.

## 5. Conclusions

In this study, we extended the parylene-C patterning method to mESCs and neuralized cells derived from mESCs. Our results illustrate that neuralized mESCs grew on serum-activated parylene-C stripes of variable widths (5–40 μm) with high conformity. Narrower stripes had a high cellular content, neurospheres and aligned neurites. Our study paves the way for establishing long-term patterned co-cultures of primary and mESC-derived neurons to engineer precise neural circuits. This is an exciting prospect for pharmacological research and investigations in fundamental neuroscience.

## Data Availability

Data are available upon request to the author.

## Supplementary Materials

The following are available online at https://www.mdpi.com/article/10.3390/ma14123174/s1, Figure S1: Assessment of the morphology and health of primary neuron and astrocyte populations. Primary cell culture examples on poly-d-lysine treated glass coverslips. Neuronal and astrocyte populations appear large and healthy. Neurons formed clusters and extended processes, whereas astrocytes expanded and covered the entire surface. Left image: 21 DIV. Right image: 7 DIV. For both images, the green channel shows GFAP (astrocytes), the red channel shows TUJ1 (neurons) and the blue channel shows DAPI (nuclei). Scale bar is 100 μm.
