# Supplementary material for: Adhesion and Growth of Neuralized Mouse Embryonic Stem Cells on Parylene-C/SiO2 Substrates
Source: Materials (Basel). 2021 Jun 9;14(12):3174. doi: 10.3390/ma14123174 (PMC8226677; doi:10.3390/ma14123174)
Supplement: Supplementary file 1 [file materials-14-03174-s001.zip › materials-1227904-supplementary.pdf]

materials

Supplementary

Adhesion and Growth of Neuralised Mouse Embryonic Stem Cells on Parylene-C/SiO_2 Substrates

Alan F. Murray [1] and Evangelos Delivopoulos [2,*]

[1] School of Engineering, University of Edinburgh, Edinburgh EH9 3JL, UK; Alan.Murray@ed.ac.uk
[2] School of Biological Sciences, University of Reading, Berkshire RG6 6AH, UK
* Correspondence: e.delivopoulos@reading.ac.uk; Tel.: +44-11-8378-8615

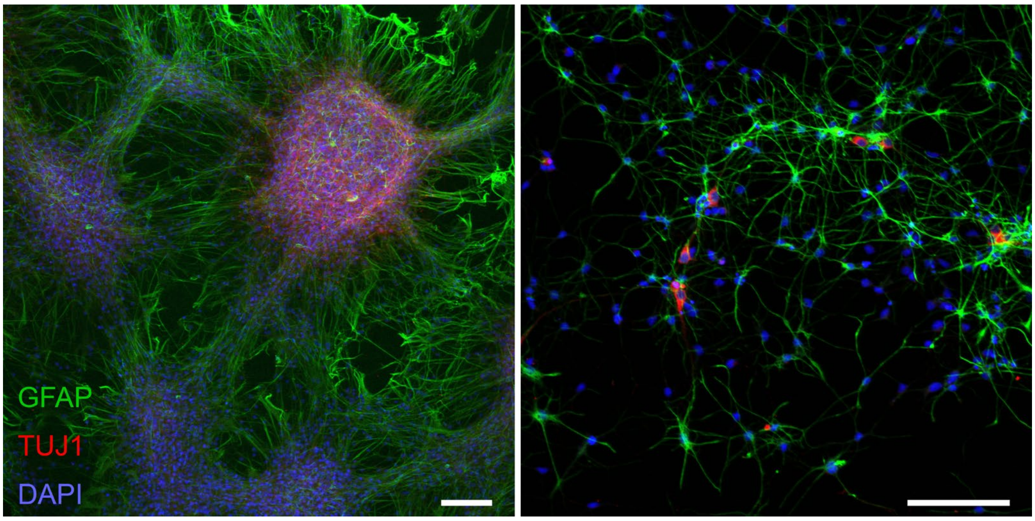

Figure S1. Assessment of the morphology and health of primary neuron and astrocyte populations. Primary cell culture examples on poly-D-lysine treated glass coverslips. Neuronal and astrocyte populations appear large and healthy. Neurons formed clusters and extended processes, whereas astrocytes expanded and covered the entire surface. Left image: 21 DIV. Right image: 7 DIV. For both images, the green channel shows GFAP (astrocytes), the red channel shows TUJ1 (neurons) and the blue channel shows DAPI (nuclei). Scale bar is 100 µm.
